# Post-traumatic rupture of the right ovary and liver after blunt abdominal trauma: A case report

**DOI:** 10.1016/j.ijscr.2019.11.044

**Published:** 2019-11-27

**Authors:** Panagiota Xaplanteri, Nada Zacharis, Charalampos Potsios, Georgios Zacharis

**Affiliations:** a School of Health Rehabilitation Sciences, Department of Nursing, University of Patras, Greece; b GP, Internal Medicine, Patras, Greece; c Department of Internal Medicine, University General Hospital of Patras, Greece; d Department of General Surgery, St. Andrew’s General Hospital, Patras, Greece

**Keywords:** Ovarian rupture, Blunt abdomen trauma, Liver rupture, Case report

## Abstract

**Introduction:**

Traumatic rupture of the ovary, due to blunt abdominal trauma, is rarely described in literature and is usually related to ovarian cyst or teratoma.

**Presentation of case:**

A 21-year-old Greek, female patient was transported to the Emergency Department complaining of abdominal pain as a result of a catapulting fall from a bicycle. It was determined that both right ovary and liver rupture had occurred. The patient was treated with rinsing of the peritoneal cavity, subhepatic packing and right ovary hemostasis.

**Discussion:**

Traumatic rupture of the ovary, due to blunt abdominal trauma, is very rare and is usually connected to former ovarian cyst or teratoma. This is the first such ever documented case in Greece.

**Conclusion:**

Although rare, injury of the ovary following blunt abdominal trauma, should always be included in the differential diagnosis of acute abdomen in female patients.

## Highlights

• Traumatic rupture of the ovary due to blunt abdominal trauma is very rare and usually connected to ovarian cyst or teratoma.
• FAST and CT-angiography missed the ovary rupture, which was revealed by the exploratory research laparotomy.
• In a hemodynamic instable patient hemostasis is performed by ovarian artery ligation through laparotomy.
• Injury of the ovary should always be included in the differential diagnosis of blunt abdomen trauma in female patients.

## 1 Introduction

Traumatic rupture of the ovary, due to blunt abdominal trauma, is rarely described in literature [1]. Presented here, is the case of a 21-year-old Greek, female patient who was transported to the Emergency Department complaining of abdominal pain as the result of a catapulting fall from a bicycle. It was determined that she was suffering from both right ovary and liver rupture. To our knowledge, this is the first such ever documented case in Greece.

The work has been reported in line with the SCARE criteria [2].

## 2 Presentation of case

A 21-year-old Greek, female patient attended the Emergency Department of our hospital complaining of abdominal pain after being catapulted and falling from her bicycle. The patient slipped and fell approximately five meters down a cliff. Her body weight landed on her bike and the handlebars impacted her abdomen forcefully. She had no previous medical history of trauma to the abdomen. Physical examination revealed abdominal pain of the right hypochondrium on palpation. She also exhibited scratches on her face and body.

On admission, her arterial blood pressure was 144/91 mmHg and her pulse rate 64/min. Laboratory findings revealed white blood cell count of 6630/mm^3^, hematocrit 35.7 %, (normal values 37–42 %), aspartate aminotransferase 993 U/L (normal values 5–40 U/L), alanine aminotransferase 1015 U/L (normal values 12–78 U/L), lactate dehydrogenase 1365 IU/L (normal values 81–230 IU/L), alkaline phosphatase 100 IU/L (normal values 50–136 IU/L), gamma-glutamyl transpeptidase 17 IU/L (normal values 5–85 IU/L).

Focused Assessment with Sonography for Trauma (FAST) scan and spiral Computed tomography of the abdomen and retroperitoneal space revealed hepatic parenchyma rupture at 5, 6, 7, and 8 segments, centrally and extending to the organ capsule at the segments 6 and 7. The exploratory research laparotomy which followed showed haemoperitoneum, large liver rupture of segments 6, 7 and active bleeding at the site of the ruptured right ovary. Blood clots were present in both the Douglas and Morison spaces. The patient was treated with rinsing of the peritoneal cavity, subhepatic packing and right ovary hemostasis. Two days later, unpacking and abdomen rinsing took place. The patient had a smooth post-operative recovery and was released without any complications.

## 3 Discussion

Parenchymal organs, which are usually injured due to blunt trauma are: liver (36 %), spleen (32 %), kidneys (24 %), small intestine (55 %), and colon (35 %) [3]. Vascular injuries to the abdomen after blunt trauma injury are less likely to occur [1]. They represent 4 % of abdominal injuries, only 7–8 % of which are due to blunt trauma [1,4]. In descending order the blood vessels mostly injured due to blunt trauma are the aorta, hepatic vein, hepatic artery, portal vein, renal artery, mesenteric blood vessel, and pelvic blood vessels [1].

Traumatic rupture of the ovary, due to blunt abdominal trauma, is very rare and it is usually connected to former ovarian cysts or teratomas [5]. So far in the literature a multi trauma patient run over by a tractor proved to suffer from ruptured cyst of the left ovary [3]. Two cases of an 18 year-old and a 15-year-old female respectively who were overrun by high-speed vehicles are also described. They proved to suffer from ovarian rupture involving a dermoid cyst [5,6]. Another case of a 45 year-old female patient involved in a car accident also proved to suffer from ovarian rupture related to ovarian teratoma [7].

Two very rare cases of ovarian artery damage, due to ovarian artery rupture, have been reported related to aneurysm and after childbirth respectively [1,8]. Our patient was catapulted from her bicycle and fell approximately five meters down a cliff. Her body weight landed on her bike and the handlebars impacted her abdomen forcefully. As a result, extensive liver damage and ovarian rupture occurred.

**Fig. 1:**
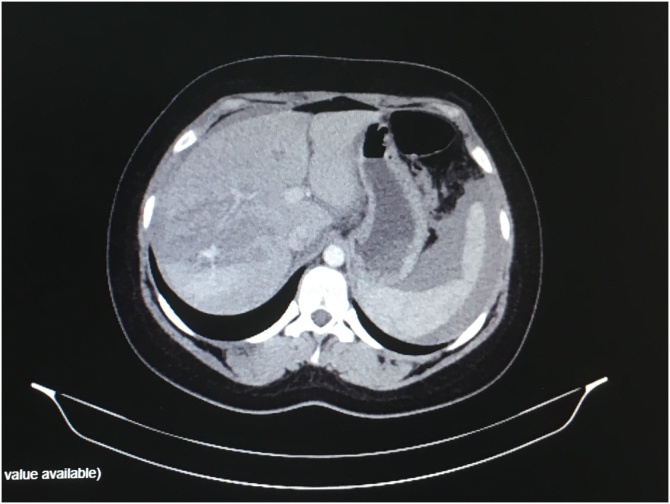
Spiral Computed tomography of the abdomen and retroperitoneal space revealed hemoperitoneum and hepatic parenchyma rupture.

Focused Assessment with Sonography in Trauma (FAST) is a worldwide accepted tool, by all medical specialties, to access trauma patients. It is a scanning protocol which provides accurate diagnoses of internal organ hemorrhage, such as hemoperitoneum, hemothorax, and hemopericardium [9]. In combination with computed tomography, angiography accurately assesses the bleeding area [1]. In our presented case, the above referred to diagnostic tools missed the ovary rupture, which was revealed by the exploratory research laparotomy (Fig. 1).

The treatment of hemorrhage, due to ovarian rupture, depends on the patients’ vital signs. In a hemodynamically stable patient selective vascular embolization is performed. In a hemodynamic unstable patient, hemostasis is performed by ovarian artery ligation through laparotomy [1]. Our patient had combined liver and ovarian rupture and was therefore treated by laparotomy where ovarian artery ligation and subhepatic packing were performed.

## 4 Conclusion

Injury of the ovary following blunt abdominal trauma, although rare, should always be included in the differential diagnosis of acute abdomen in female patients.

## Sources of funding

This research was supported by funding of the Department of General Surgery St. Andrew’s General Hospital, Patras, Greece.

## Ethical approval

The study has been approved by the Ethics committee of St. Andrew’s General Hospital, Patras, Greece, approval number 37117.

## Consent

Written informed consent was obtained from the patient for publication of this case report and accompanying images. A copy of the written consent is available for review by the Editor-in-Chief of this journal on request”.

## Author’s contribution

All authors have contributed in study concept and design, data collection, data analysis and interpretation. Panagiota Xaplanteri has written the paper.

## Registration of research studies

N/A.

## Guarantor

All authors.

## Provenance and peer review

Not commissioned, externally peer-reviewed.

## Declaration of Competing Interest

The authors declare that they have no competing interests.

## References

[1] Kang J.Y., Han M.S., Choi I.J., Ann H.S., Cha M.S. (2012). A case of retroperitoneal hematoma caused by left ovarian artery rupture following mild blunt trauma. Korean J. Obstet. Gynecol..

[2] Agha R.A., Borrelli M.R., Farwana R., Koshy K., Fowler A., Orgill D.P., For the SCARE Group (2018). The SCARE 2018 statement: updating consensus Surgical CAse REport (SCARE) guidelines. Int. J. Surg..

[3] Madej B., Fijalkowski P., Burdan F. (2008). Post-traumatic rupture of the ovarian cyst—case report. Pol. Przegl. Chir..

[4] Asensio J.A., Forno W., Roldan G., Petrone P., Rojo E., Ceballos J. (2002). Visceral vascular injuries. Surg. Clin. North Am..

[5] Kimbrell B., Emami C., Petrone P., Asensio J. (2007). Ruptured ovarian cystic teratoma secondary to blunt abdominal trauma: a very unusual case. J. Trauma: Inj. Infect. Crit. Care.

[6] Yohann A., Lee C.W., Islam S. (2017). Traumatic rupture of an ovarian teratoma. Case report and review of the literature. J. Pediatr. Surg. Case Rep..

[7] Haidar M.G., Sharaf N.A. (2018). A rare presentation of traumatic rupture teratoma in multi-traumatic adult patient diagnosed and excised laparoscopically: a case report. J. Univ. Surg..

[8] Guillem P., Bondue X., Chambon J.P., Lemaitre L., Bounoua F. (1999). Spontaneous retroperitoneal hematoma from rupture of an aneurysm of the ovarian artery following delivery. Ann. Vasc. Surg..

[9] Pace J., Arntfield R. (2018). Focused assessment with sonography in trauma: a review of concepts and considerations for anesthesiology. Can. J. Anaesth..

